# Precise colocalization of sorghum’s major chilling tolerance locus with * Tannin1* due to tight linkage drag rather than antagonistic pleiotropy

**DOI:** 10.1007/s00122-023-04534-4

**Published:** 2024-02-03

**Authors:** Anthony Schuh, Terry J. Felderhoff, Sandeep Marla, Geoffrey P. Morris

**Affiliations:** 1 Department of Soil and Crop Science, Colorado State University, Fort Collins, CO 80526 USA; 2 Department of Agronomy, Kansas State University, Manhattan, KS 66506 USA

## Abstract

Chilling tolerance in crops can increase resilience through longer growing seasons, drought escape, and nitrogen use efficiency. In sorghum (*Sorghum bicolor* [L.] Moench), breeding for chilling tolerance has been stymied by coinheritance of the largest-effect chilling tolerance locus, *qSbCT04.62*, with the major gene underlying undesirable grain proanthocyanidins, WD40 transcriptional regulator *Tannin1*. To test if this coinheritance is due to antagonistic pleiotropy of *Tannin1*, we developed and studied near-isogenic lines (NILs) carrying chilling tolerant haplotypes at *qCT04.62*. Whole-genome sequencing of the NILs revealed introgressions spanning part of the *qCT04.62* confidence interval, including the *Tannin1* gene and an ortholog of Arabidopsis cold regulator *CBF/DREB1G*. Segregation pattern of grain tannin in NILs confirmed the presence of wildtype *Tannin1* and the reconstitution of a functional MYB-bHLH-WD40 regulatory complex. Low-temperature germination did not differ between NILs, suggesting that *Tannin1* does not modulate this component of chilling tolerance. Similarly, NILs did not differ in seedling growth rate under either of two contrasting controlled environment chilling scenarios. Finally, while the chilling tolerant parent line had notably different photosynthetic responses from the susceptible parent line – including greater non-photochemical quenching before, during, and after chilling – the NIL responses match the susceptible parent. Thus, our findings suggest that tight linkage drag, not pleiotropy, underlies the precise colocalization of *Tan1* with *qCT04.62* and the *qCT04.62* quantitative trait nucleotide lies outside the NIL introgressions. Breaking linkage at this locus should advance chilling tolerance breeding in sorghum and the identification of a novel chilling tolerance regulator.

**Supplementary Information:**

The online version contains supplementary material available at 10.1007/s00122-023-04534-4.

## Introduction

Improving crop adaptation to climatic stressors, such as drought, heat, and cold, is important to safeguard agricultural productivity and food security (Maggio et al. 2015). Development of more chilling tolerant crops can improve agricultural sustainability by increasing crop yields by facilitating early planting, increasing yield through a lengthened growing season, and better-aligning crops’ evapotranspirative needs with precipitation and temperature patterns (Long and Spence 2013; Raymundo et al. 2021). Early planting of chilling tolerant crops can also minimize nitrogen loss via runoff and emissions during fallow periods (Mosier et al. 1998).

**Fig. 1:**
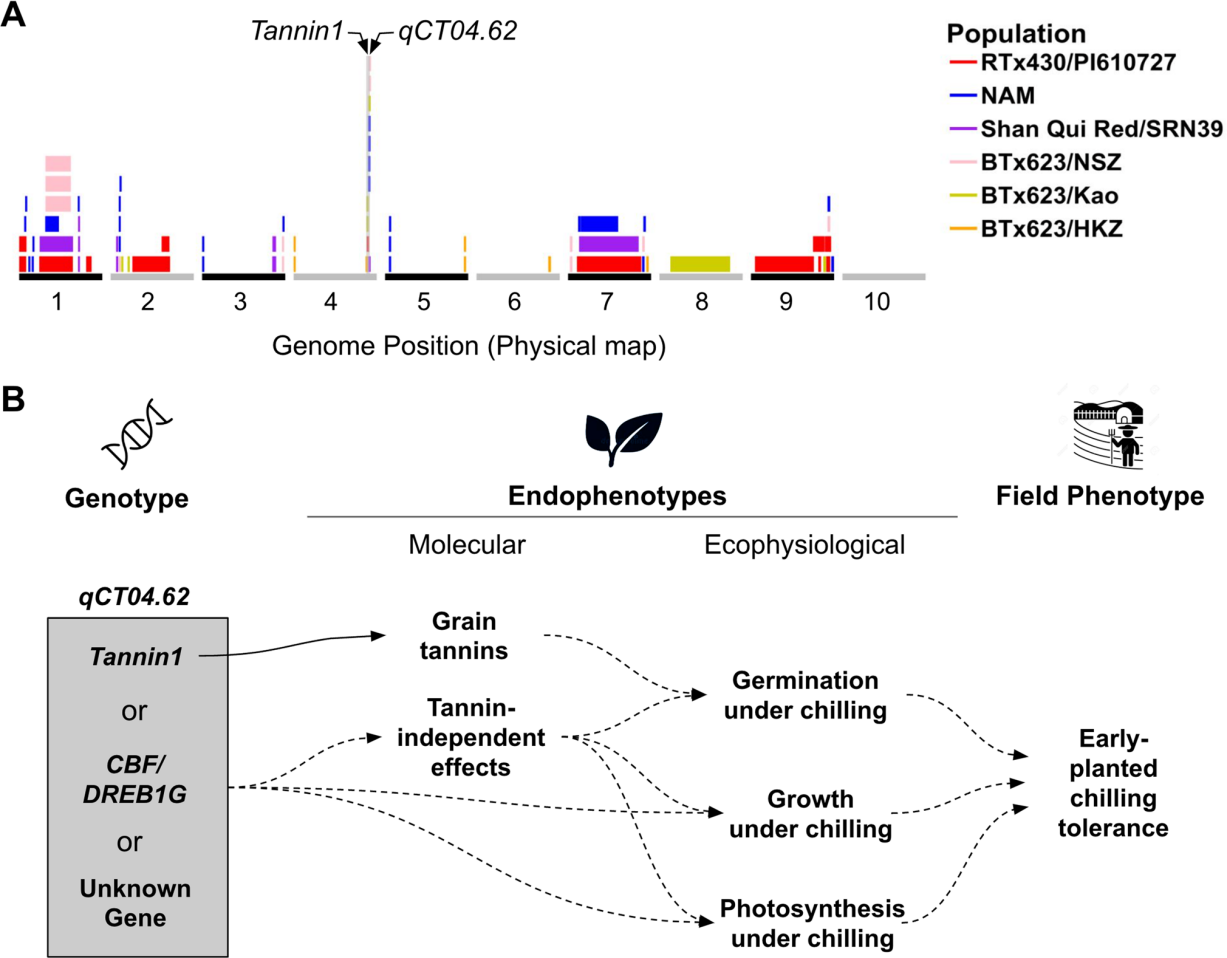
Previous observations on *qCT04.62* and hypotheses on the relationship of *Tannin1* and other genes to chilling tolerance. **A** QTL from NAM and biparental mapping studies for cold tolerance in sorghum (Knoll et al. 2008; Burow et al. 2011; Marla et al. 2019). QTL positions are from the Sorghum QTL Atlas (Mace et al. 2019). Note, all six studies listed precisely identified *qCT04.62*. **B** Graphical hypotheses on which gene underlies *qCT04.62* (*Tannin1*, *CBF/DREB1G*, or an unknown gene) and the mechanism by which *qCT04.62* leads to early-planted chilling tolerance. Solid lines indicate established knowledge and dashed lines indicate hypotheses to be tested

Sorghum is a tropical-origin crop that is important for agricultural sustainability. It is the fourth most produced cereal crop, particularly in semiarid environments (Monk et al. 2014). Historically, breeding for chilling tolerance in sorghum has been unsuccessful. There is little chilling tolerance variation in commercial US breeding programs, which has prompted a > 60-year search for chilling tolerance in exotic germplasm, particularly kaoliang landraces from northern China (Stickler et al. 1962; Franks et al. 2006). Multiple chilling tolerance mapping studies in sorghum have shown a complex genetic architecture (Knoll et al. 2008; Burow et al. 2011; Ortiz et al. 2017; Moghimi et al. 2019). Joint linkage mapping (JLM) in large nested association mapping (NAM) population further identified several co-localizations between chilling tolerance (vigor and emergence) QTL and genes regulating grain tannins (*Tannin1* and *Tannin2)* and height (*Dw1* and *Dw3*) (Marla et al. 2019). In particular, *qSbCT04.62* (hereafter *qCT04.62*), a major effect chilling tolerance QTL identified in many studies, co-localizes precisely with *Tannin1*, a canonical grain tannin regulator (Wu et al. 2012), in at least six studies (Fig. 1A). The peak SNP from JLM is less than 50 kb from the *Tannin1* gene, and near an ortholog of CBF, suggesting that the chilling sensitive allele at *qCT04.62* is either a pleiotropic effect of *Tannin1* loss-of-function or a variant at the nearby *CBF/DREB1G* (Fig. 1A). Unfortunately, the colocalization is antagonistic, causing grain tannins, a commercially unacceptable trait, to be co-inherited with the chilling tolerance allele.

*Tannin1* is a sorghum ortholog of Arabidopsis *TTG1* (Wu et al. 2012), the WD40 subunit of the MYB-bHLH-WD40 (MBW) regulatory complex, and a master regulator of epidermal traits (Tian and Wang 2020). *TTG1* is known to be a major pleiotropic regulator of flavonoid biosynthesis, root hair and trichome development, and the presence of seed coat mucilage. It is unknown if *Tannin1* shares any of these functions beyond the regulation of seed proanthocyanidins and what other pleiotropic effects *Tannin1* might have on commercial sorghum cultivars besides promoting grain tannins. Coinheritance of chilling tolerance and grain tannins in sorghum could be explained by either of two competing hypotheses, each with different consequences for breeding. If the coinheritance is caused by linkage between *Tannin1* and the *qCT04.62* quantitative trait nucleotide (QTN), then it is possible through recombination to break the linkage and use the trait for breeding. Alternatively, if coinheritance is due to pleiotropic control of both traits by *Tannin1,* the allele will only be usable for chilling tolerance in commercial grain sorghum if the nontannin phenotype is conferred by other genes.

Near isogenic lines (NILs) are powerful tools in genetics research, particularly when paired with high-resolution genomic data (Zhang 2021). To test these hypotheses, we created NILs using MAS to introgress part of *CT04.62* + (*pCT04.62*) originating from Chinese sorghum line Hong Ke Zi (HKZ), including *Tannin1* and *CBF/DREB1G*, into a chilling sensitive BTx623 background, replacing the loss-of-function *tan1-b* allele (Wu et al. 2012). We used the NILs for genetically-controlled experiments to eliminate hypotheses (Platt 1964) on the mechanisms underlying *qCT04.62* and elucidate the genotype to phenotype relationship for chilling tolerance (Fig. 1B). Based on the lack of differential response of NILs to germination, seedling growth, and photosynthesis under chilling stress, we conclude that (1) the colocalization of *Tannin1* and *qCT04.62* is most likely due to tight linkage drag, not antagonistic pleiotropy and (2) the QTN for *qCT04.62* likely lies outside the introgression region (and not in *CBF/DREB1G*) in an unknown novel chilling tolerance regulator.

## Results

### NIL pairs are heterogeneous at qCT04.62, including Tannin1 and CBF, but homogeneous through most of the genome

**Fig. 2:**
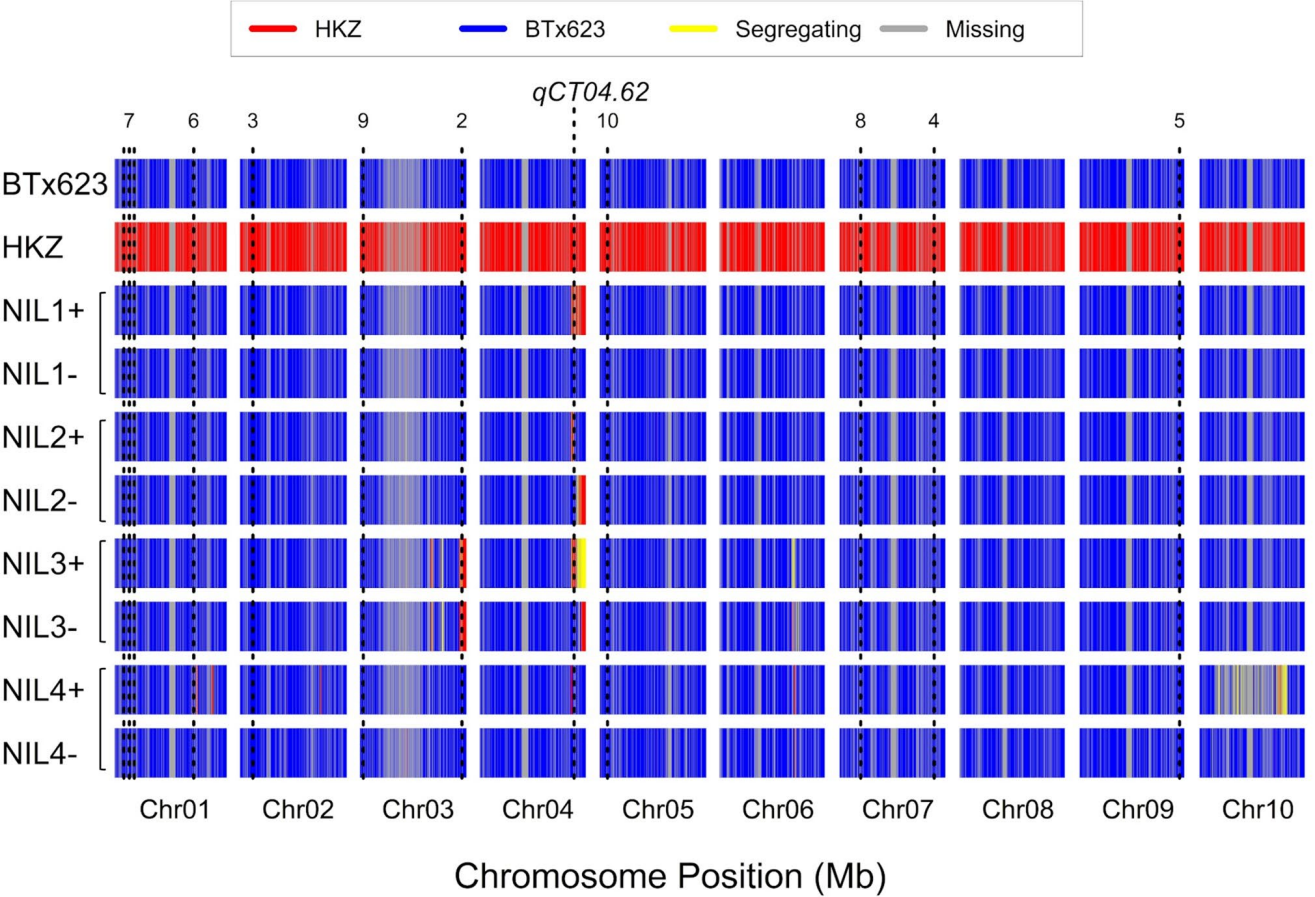
Whole genome analysis of NILs selected for and against HKZ haplotype at *qCT04.62* confirms the precision of the introgression. Sliding window scan of low coverage genotype data at 10 kb resolution. Visualization is of alternate allele number relative to HKZ for two individuals per genotype. Red indicates HKZ allele (alternate^x^ /alternate^HKZ^ >  = 0.2), blue indicates BTx623 allele (alternate^x^ /alternate^HKZ^ < 0.2), yellow indicates when the genotype call differs between replicates, and gray indicates missing data. Dotted lines are chilling tolerance QTL (Marla et al 2019; Table S1). Haplotypes are inferred using a genome wide average of 3500 biallelic SNPs per MB (Fig. S1)

To characterize introgression of *qCT04.62* from HKZ into the BTx623 background, we used low-coverage whole-genome resequencing of positive and negative NILs for each family. After filtering for high-quality biallelic SNPs, a genome-wide average of 3500 SNPs per Mb was used in genotyping across NILs and parental lines (Fig. S1). In NIL 1, 2, and 3 families, there is < 5% genomic segregation between biological replicates, and also, under 5% of the total genome originates from HKZ outside the introgressions (Fig. 2). In the NIL4 family, one NIL4 + individual appears heterozygous for most of chromosome 10 (Fig. 2). To test whether any QTL besides *qCT04.62* are also segregating between the NIL pairs, we checked for colocalization of introgressions with previously identified chilling tolerance QTL (Marla et al. 2019; Fig. 2). Besides a chromosome 10 in NIL4 + , there appear to be few HKZ introgressions in the NILs, and none overlapping with CT QTL, with the exception of the NIL3 family, where one small introgression on chromosome 3 spans the early season emergence QTL *qEPEC.3–72* in both NIL + and NIL- individuals (Fig. 2, Table S1).

**Fig. 3:**
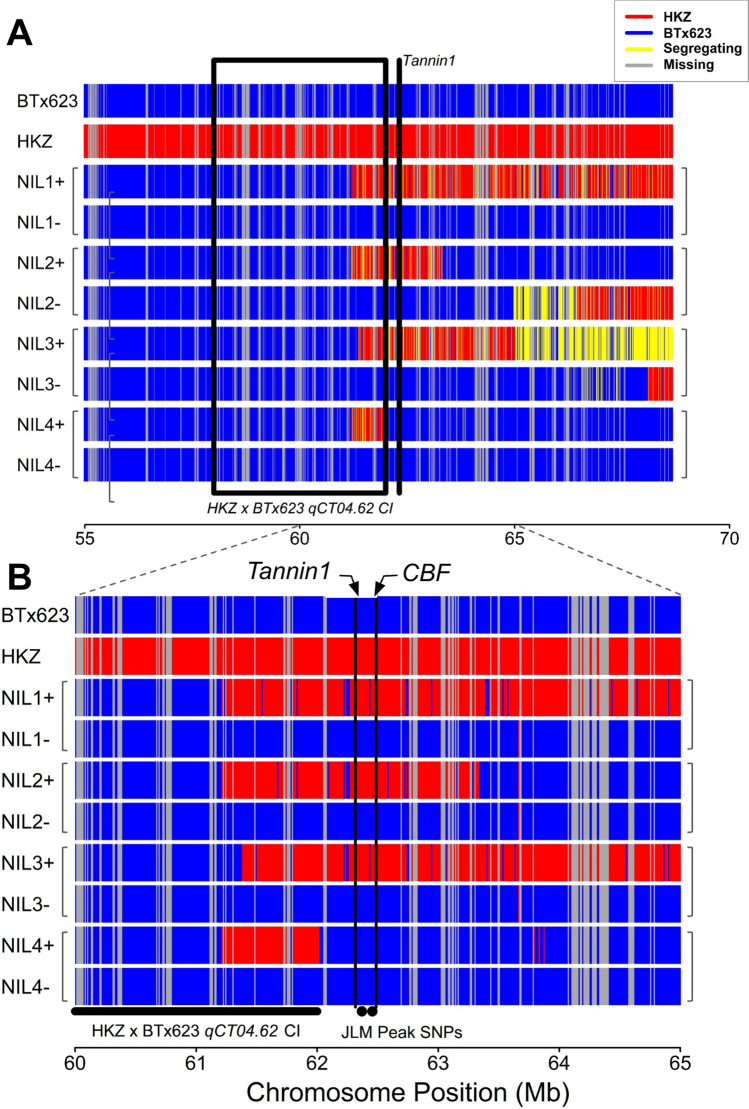
Targeted haplotype analysis of NILs at *qCT04.62* confirms the introgression of peak association for *qCT04.62*, *Tannin1*, and *CBF/DREB1G*. **A** High-resolution view of *qCT04.62* and introgression site on chromosome 4, 55–65 Mb. *Tannin1* location is denoted by black line. Confidence interval for *qCT04.62* in the HKZ NAM family is denoted by a black box. **B** High-resolution view of the region surrounding *Tannin1*. Black lines are genes (*CBF* and *Tannin1*) and dots are peak associations for *qCT04.62* from joint linkage mapping (JLM) in a nested association mapping population (NAM) (Marla et al. 2019). The *qCT04.62* confidence interval (CI) from single linkage mapping in the HKZ ✕ BTx623 family within the NAM population is denoted by the horizontal black bar. Haplotypes are inferred from 2000 to 5000 biallelic SNPs per Mb (Fig. S1)

Genomic regions introgressed from the donor parent into NIL1 + , NIL2 + , and NIL3 + range in size from 3–15 Mb. The introgression encompassed peak SNPs from chilling tolerance JLM (S4_62368531, S4_62455479) and ~ 25% of *qCT04.62* confidence interval from single linkage mapping in the HKZ ✕ BTx623 NAM family (Fig. 3A). Notably, the introgressions in NIL1, NIL2, and NIL3 include both *Tannin1* (Sobic.004G280800, Chr04: 62,315,396) and a sorghum *CBF* ortholog (Chr04: 62,486,634) of rice chilling tolerance regulator *OsDREB1G* (Sobic.004G283201) (Moon et al. 2019). NIL 4 + has a smaller introgression of about 1 Mb, which also includes ~ 25% of *qCT04.62* CI but lacks *Tannin1*, JLM peak SNPs, or the *CBF* ortholog (Fig. 3B). NIL families 1–3 segregate for functional/non-functional alleles of *Tan1* between the positive and negative lines, while the NIL4 family appears fixed for *tan1-b*. Overall, the segregation patterns of all NIL families establish their usefulness for testing the *Tan1* linkage vs. pleiotropy hypotheses.

**Fig. 4:**
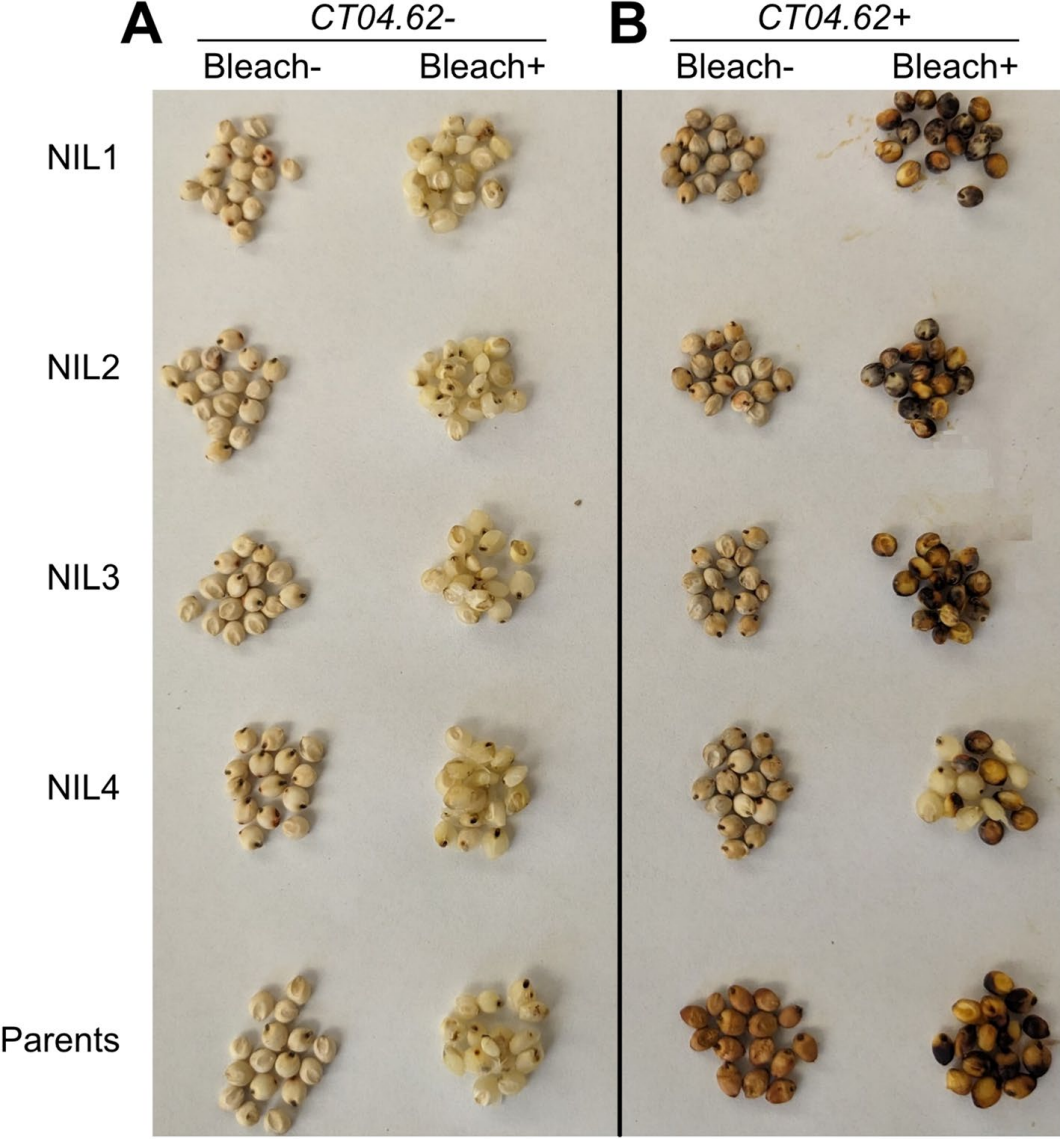
Bleach test confirms the presence of wildtype *Tan1* and a functional MYB-bHLH-WD40 regulatory complex in introgression lines. NILs are organized vertically by family with parent lines as controls at bottom. QTL ± denotes selection for *CT04.62*^+^ or *CT04.62*^*−*^ in NIL families and the allele itself in parents (Left: BTx623, nontannin; Right: HKZ, tannin). Tannin containing seeds darken when soaked in NaOH/bleach solution, while nontannin seeds become white/yellow

To validate the genomic findings and test for homozygosity at *Tan1*, a bleach test was used to test for the presence of grain tannins (Fig. 4). As expected, all NIL- lines were tannin deficient and fixed for the *tan1-b* allele. In NIL1-3 + , tannins were present in all seeds, consistent with genotypic predictions. In NIL4 + , we expected fixation for the *tan1-b* allele but observed segregation for tannin + /tannin- seeds. As all NIL4 + seeds originated from a single-selfed plant, this suggests heterozygosity at *Tannin1* in NIL4 + ’s direct progenitor. This finding prompted us to exclude the NIL4 family from further experimentation to avoid confounding effects from segregating *Tannin1* alleles.

### The partial introgression of qCT04.62 including Tan1 does not regulate low-temperature germination

**Fig. 5:**
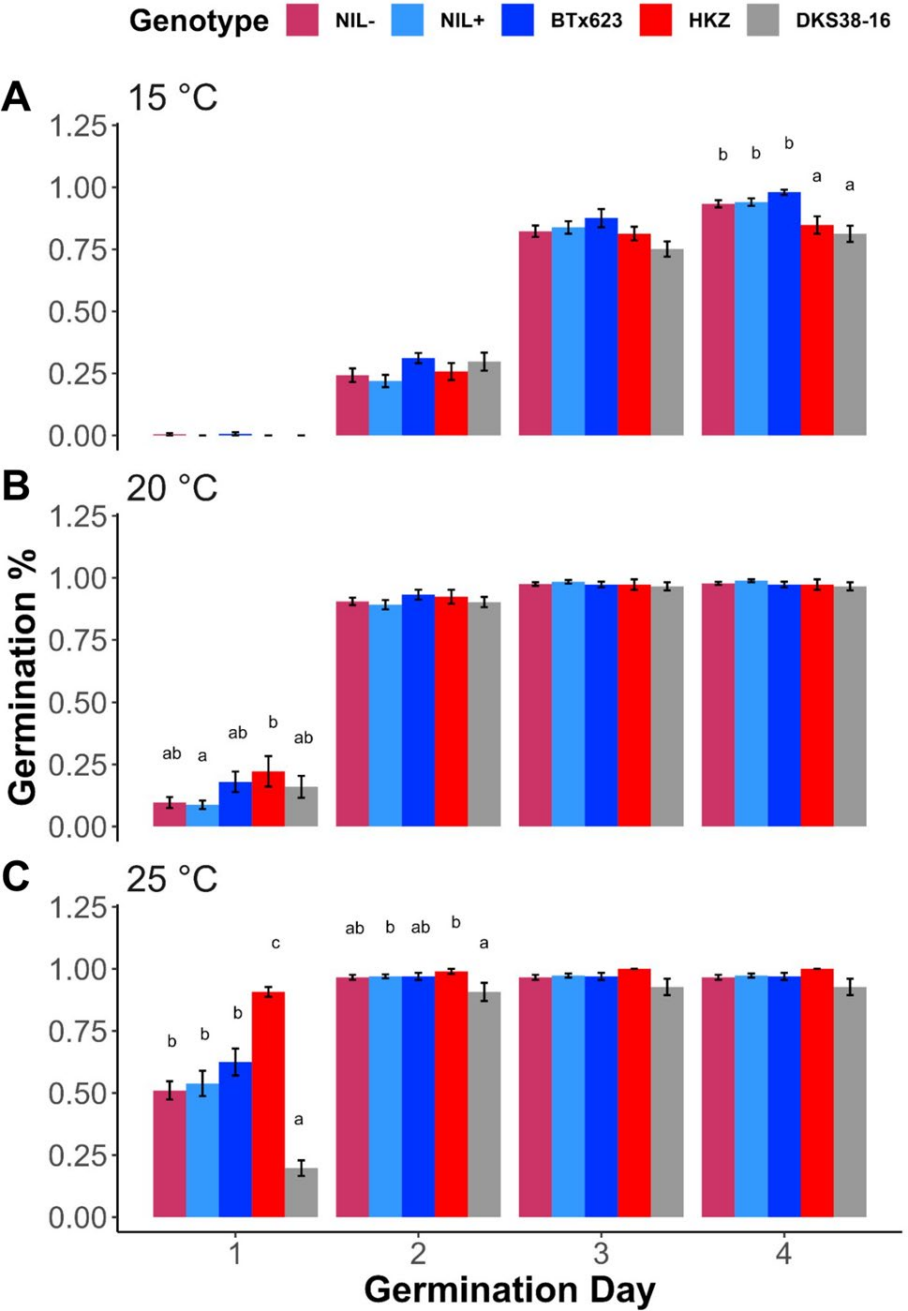
Effects of mild chilling on germination do not differ between NILs. (A) Germination rate at 15 °C (Day 3 ***), (B) at 20 °C (Day 1 *), and (C) at 25 °C (Day 1 ***, Day 2 *) for NILs, parent lines, and a commercial check. NIL + and NIL- are means from NIL1 and NIL2 families. DKS38-16 is a commercial hybrid used as a positive control for germination. Error bars span ± one standard error Significance calculated using one-way ANOVA. Pairwise comparisons at significance level *p* < 0.05 were made using Tukey HSD with different letters representing statistical difference between genotypes. Significance codes: *p* < 0.001 = ***, *p* < 0.01 = **, *p* < 0.05 = *

Mapping studies showed that *qCT04.62* regulates early season emergence in the field (Knoll et al. 2008; Burow et al. 2011; Marla et al. 2019) as well as low-temperature germination (Fig. 1A), suggesting that wildtype *Tan1* confers low-temperature germination (Fig. 1B). To test this hypothesis, we conducted germination tests in controlled laboratory conditions in BTx623, HKZ, and the NIL1 and NIL2 families. (The NIL3 family was excluded from this test to avoid possible confounding effects from introgression of *qEPEC.3–72*.) At the lowest temperature (15 °C) there was no germination on day one and no significant genotypic differences on days two and three (*p* > 0.05). By day 4, there is a highly significant genotypic effect (25%; *p* < 10^−4^) with HKZ and DKS38-16 having lower overall germination after four days than BTx623 or the NILs (Fig. 5A). At 20 °C, there is a significant genotypic effect on day one (*p* = 0.01), with HKZ having a higher germination rate than BTx623, NILs, or DKS38-16 (BTx623: 16%, NILs: 8%, HKZ: 25%). Again, the NILs group together. Further, no genotypic differences between lines during days 2–4 (Fig. 5B; *p* = 0.5–0.8). At 25 °C there is a significant genotype effect on days one and two (*p* < 10^−4^), with HKZ having 25% greater germination (Fig. 5C). The slower germination of HKZ at 15 °C combined with faster germination at higher temperatures suggests genotypic control of temperature-dependent germination in HKZ and BTx623.

### pCT04.62/Tan1 introgression does not regulate growth under chilling in controlled environment

**Fig. 6:**
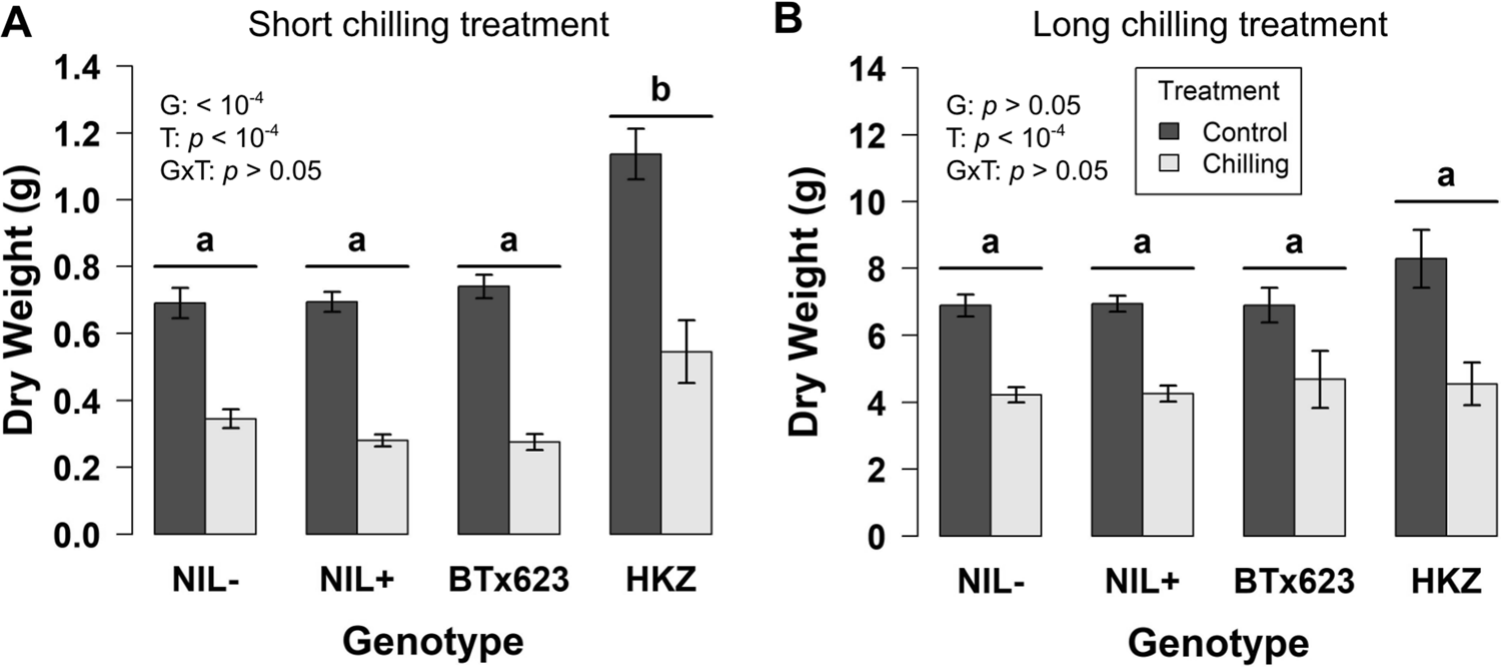
Reduction of growth due to chilling is similar across NILs and parent lines. **A** Effects of short chilling treatment and genotype on growth, with 1-week-old seedlings subjected to three-day chilling shock and seven-day recovery. **B** Effects of long chilling treatment and genotype on growth, with 5-day-old seedlings subjected to month long chilling treatment. Error bars span ± one standard error. NIL data are means of families 1–3. The *p*-values were calculated using two-way ANOVA. Pairwise comparisons were made using Tukey HSD

To better understand the role of chilling tolerance in early planted vigor and to test the hypothesis that we introgressed the causal factor underlying the regulation of chilling tolerance by *qCT04.62* (Fig. 1B), we subjected NILs and parents to testing under controlled environment chilling conditions. A three-day cold shock treatment with a week-long recovery resulted in significant treatment and genotype effects (*p* < 10^−4^,* p* < 10^−4^), but not GxT (Fig. 6A; *p* = 0.07). Similarly, during month-long temperature treatments (Fig. 6B), all lines exhibit significant treatment effects (*p* < 10^−4^) but lack significant genotype or genotype by treatment (GxT) effect for dry weight (*p* = 0.3, *p* = 0.5). However, HKZ has a slightly higher mean weight (1.5 g) than other lines, though this is non-significant (Fig. 6B; *p* = 0.1–0.3). The genotype effects are driven primarily by more vigorous growth during warm temperature phases for HKZ plants under both chilled and controlled conditions. Overall, HKZ has a faster growth rate compared to BTx623 or NILs (33%) but does not exhibit a GxT effect.

### Chilling susceptible and tolerant parent lines have contrasting photosynthetic response to chilling, but pCT04.62/Tan1 NILs mirror the susceptible parent

**Fig. 7:**
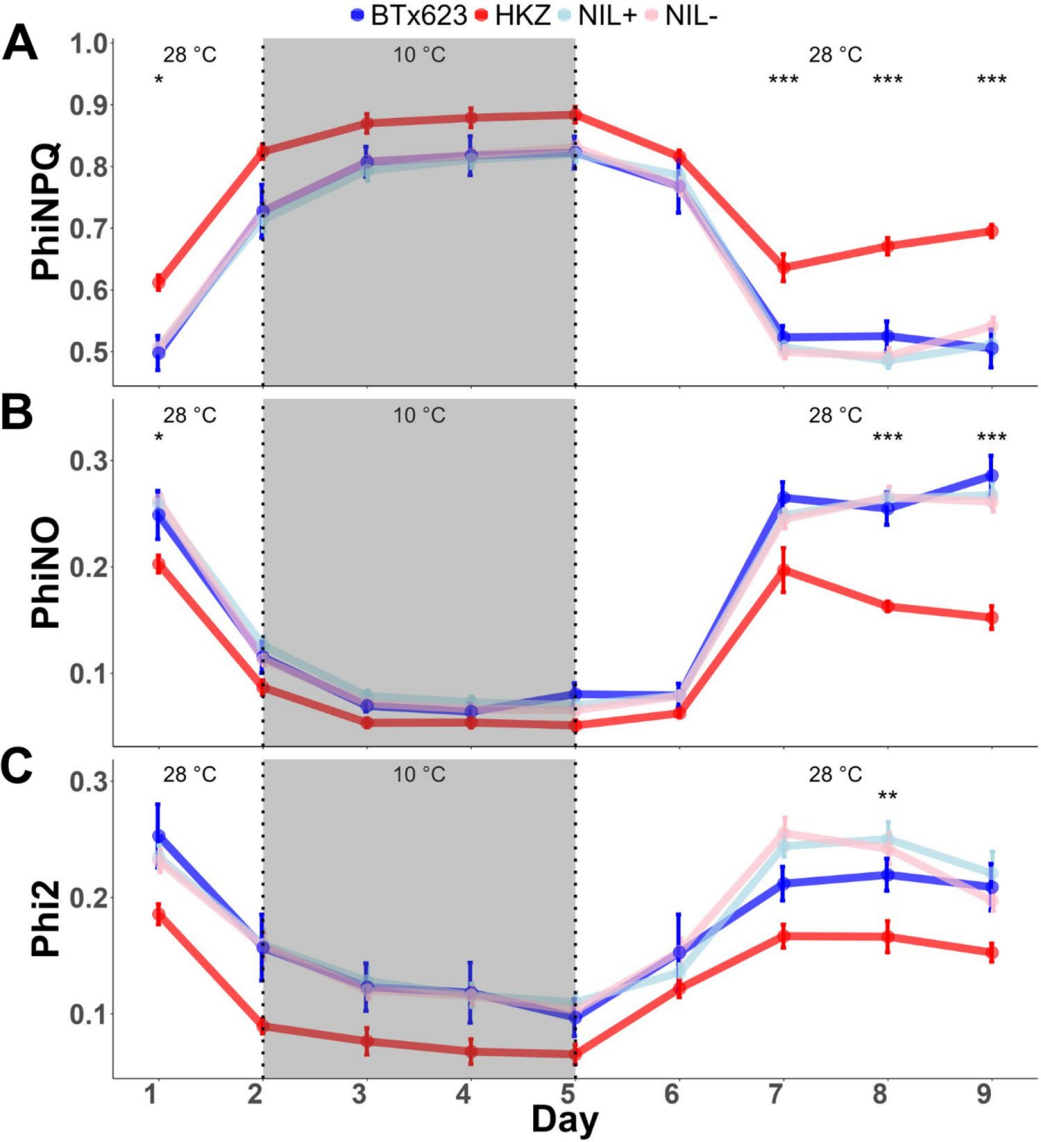
Photosynthetic parameters before, during, and after chilling are similar for NILs and the chilling susceptible parent line BTx623, but distinct from the chilling tolerant parent HKZ. Shaded area represents chilling conditions at 10 °C while non-shaded area represents control conditions at 28 °C. **A** PhiNPQ, **B** PhiNO, and (**C**) Phi2 measurements were taken simultaneously using a MultiSpeQ fluorometer. NIL data are means across families 1–3. Significant difference among genotypes is measured by one-way ANOVA for each day independently and is denoted: * *p* < 0.05, ** *p* < 0.01, *** *p* < 0.001

To test the hypothesis that *pCT04.62/Tan1* governs chilling tolerance through the regulation of NPQ, plants were subjected to a time course analysis of photosynthetic parameters under controlled-environment chilling stress. The photochemical function was fluorometrically tracked for significant differences across genotypes at the same time points. Phi2 is the realized steady state efficiency of photosystem II and reflects the yield of light energy successfully used for photosynthesis while PhiNPQ reflects the dispersal of excess light energy as heat, and PhiNO reflects non-regulated light energy dissipation, a central driver of photodamage (Kuhlgert et al. 2016). The baseline for each photosynthetic measurement was established day one before chilling stress (Fig. 7A-C). There were significant differences among genotypes for PhiNPQ and PhiNO (Fig. 7A-B). Under chilling, all genotypes exhibit increased NPQ, measured as PhiNPQ, with HKZ exhibiting consistently higher levels than BTx623 across all days, with statistical significance at days seven, eight and nine post-chilling (*p* < 10^−4^ on each day). Further, BTx623 returned to baseline during the recovery period, while HKZ became elevated from baseline on days eight and nine, suggesting a post-chilling reaction to chilling stress in HKZ.

To test whether genes in *pCT04.62* + (including *Tannin1* and CBF) contributed to the differences in photosynthetic response between the chilling susceptible and tolerance parent lines, we next compared photosynthetic parameters in the NILs. Both NILs grouped with BTx623 across all days and did not exhibit the elevated photoprotection seen in HKZ (Fig. 7A). The PhiNO results reinforces the trends uncovered when measuring NPQ. Across all days, HKZ sustains the lowest levels of PhiNO, while both NILs grouped with BTx623 with statistical significance between HKZ and BTx623 on days one, eight, and nine (-20%, -30%, -45%; *p* = 0.02, *p* < 10^−4^, *p* < 10^−4^). On day one, HKZ shows relatively lower levels of PhiNO. During chilling, all lines have decreased PhiNO, corresponding with increases in NPQ, with HKZ exhibiting the lowest levels. During recovery on days 6–9, PhiNO for BTx623 and the NILs are slightly elevated from baseline, while HKZ drops below baseline. This data also suggests that HKZ sustains decreased levels of photodamage at all points of the time course analysis (Fig. 7B). Interestingly, total input into photosynthesis, measured as Phi2, was lower in HKZ relative to BTx623 and NILs (0.05–0.1) with statistical significance on day 8 (*p* = 0.001). Both NIL lines are consistently grouped with BTx623. Phi2 dramatically decreased in all lines under chilling, with HKZ at about half the rate of BTx623 (Fig. 7C). Overall, HKZ shows a more conservative photosynthetic strategy that prioritizes protection over maximum photosynthetic capacity, a trend which is amplified upon exposure to chilling stress.

## Discussion

### *Coinheritance of Tan1 and CT04.62* + *due to tight linkage not antagonistic pleiotropy*

Mendelization is an effective genetics strategy for mapping genotype to phenotype in complex traits (Zhang 2021). In this study, we developed and used NILs to test whether the precise colocalization of *Tannin1* and *qCT04.62* (Knoll et al. 2008; Burow et al. 2011; Marla et al. 2019) (Fig. 1A) is due to (a) pleiotropic effects of *Tannin1,* (b) tight linkage of *Tannin1* with a putative conserved cold tolerance gene (*CBF/DREB1G*), or (c) tight linkage with an unknown chilling tolerance gene (Fig. 1B). Whole-genome resequencing of the NILs (Fig. 3) revealed very little segregation between lines, which allowed us to isolate the effects of *pCT04.62* + */Tan1* and exclude lines from experiments with off-target introgressions that could have confounding effects from other QTL (e.g., NIL3 family from germination tests). From the genomic data, we could also identify the size of on-target introgressions in NIL + and examine what pertinent genetic features are included (Fig. 4). This detailed genomic assessment already allows us to begin excluding specific candidate genes and is a starting point for continued fine mapping at this locus. With the NILs, we were also able to Mendelize *pCT04.62* + */Tan1* to run controlled genetic experiments on the introgressions function in chilling tolerance (Figs. 2–3).

Our study found no effect of alternate *Tannin1* alleles on chilling tolerance, suggesting *Tan1* is linked with the *qCT04.62* chilling tolerance QTN. The NIL families used in chilling tolerance testing were segregating for grain tannins and *Tannin1* alleles, which had no measurable effect on germination, vigor, or photosynthesis (Figs. 5–7). If the association between *Tan1* and chilling tolerance is truly a linkage, the result is advantageous from a breeding perspective, as the respective alleles can be recombined to break the cosegregation of the two traits, unlocking the potential to use *qCT04.62* as a target for marker assisted selection.

Another hypothesis we tested is whether variation in *CBF/DREB1G* within the introgression underlies chilling tolerance at *qCT04.62*. In Arabidopsis, the CBF regulon is a major cold acclimation regulator and functions through JA upregulation of CBF genes (Fowler and Thomashow 2002; Hu et al. 2017). In sorghum, chilling upregulated CBF (Marla et al. 2017), and a CBF ortholog precisely colocalized with *qCT04.62* (Fig. 1A, 3B) (Marla et al. 2019). This CBF gene is an ortholog with known rice chilling tolerance regulator *OsDREB1G* and maize CBF *ZmDREB1.9* (Liu et al. 2013; Moon et al. 2019). Given that *CBF/DREB1G* was in the introgressions (Fig. 3B), our findings suggest that this gene does not underlie *qCT04.62*. In this study, we found *pCT04.62* + to have no apparent regulatory effect on chilling tolerance (Figs. 5–7), allowing us to rule out genes in this region as the functional regulators of the QTL. This information provides a foundation to build on for future fine mapping and functional genetic experiments.

### *The QTN that underlies HKZ’s qCT04.62 chilling tolerance likely lies beyond the pCT04.62* + *introgressions*

With the introgression of the NAM peak SNPs and functional *Tannin1*, we expected NIL + lines to exhibit chilling tolerance (Fig. 1). Unexpectedly, in all assays performed, NIL + showed no evidence for induction of a response indicative of chilling tolerance, though we did see differing chilling responses between BTx623 and HKZ, the positive and negative controls for chilling tolerance (Figs. 5–7). HKZ exhibits more rapid germination and initial growth than BTx623, but surprisingly, in response to chilling stress, HKZ seems to have reduced growth, germination, and photosynthesis. This trade-off of growth may be a protective mechanism that allows the plant to avoid exacerbating stress under poor growing conditions. In the case of photosynthesis, this strategy may lead to a reduction in photosynthetic input and a reduction in photodamage (Taylor and Rowley 1971; Ortiz et al. 2017). These observations show that chilling tolerant HKZ employs different responses to chilling than susceptible BTx623, though it is difficult to make a conclusion on the mechanism.

While HKZ displayed phenotypes suggestive of chilling tolerance for vigor, germination, and photosynthesis, NIL + and NIL- lines, which are in BTx623 genetic background, behave nearly identically to each other and BTx623 (Figs. 5–7). This result is unexpected if the QTN was within *pCT04.62* + */Tan1*, since *qCT04.62* underlies 17% of the phenotypic variation in the HKZ family in field trials for early season vigor (Marla et al. 2019). These conclusions are further strengthened by the genotyping data, which shows that only 25% of the *qCT04.62* HKZ confidence interval was introgressed with *pCT04.62* + */Tan1* (Fig. 3A), which makes it possible that the QTN driving chilling tolerance regulation was not included in the introgression leaving all NIL lines homozygous for the BTx623 allele. If *pCT04.62* + */Tan1* introgression does not contain the QTN, this would indicate that the functional *Tan1* allele is not contributing to the chilling tolerance phenotype at *qCT04.62*, and the association is caused by a linkage instead of pleiotropy. Previous studies found *Tannin2* to be associated with chilling tolerance (Marla et al. 2019), strengthening the hypothesis that either grain tannins or the MBW complex play a role in chilling tolerance regulation (Fig. 1B). Genetic data supporting this hypothesis is mixed, as the peak JLM SNPs precisely co-localized with *Tannin1*, while the HKZ confidence interval was located upstream (Fig. 5).

It is also possible that the chilling tolerance QTN was introgressed, but there is another explanation for the lack of GxE for germination, growth, and photosynthesis under chilling (Figs. 5–7). Potentially, the *qCT04.62* QTN acts epistatically with another QTL that was not introgressed, rendering *CT04.62* + non-functional in the NILs (Reif et al. 2009; Bekele et al. 2014). Alternatively, the chilling response may be regulated by the QTN but the phenotypic response is outside the growth and photosynthesis measurements used in this study. Also, the controlled environment stress used in this assay may be insufficient to elicit the chilling tolerance mechanism regulated by *CT04.62* under early-planted field conditions. Natural environments are extremely temporally variable for temperature and light, two critical factors in chilling stress (Taylor and Rowley 1971). The experimental light intensity (700 μmol m^−2^ s^−1^) may have been insufficient to provide adequate stress, compared to natural sunlight, which can exceed 1500 μmol m^−2^ s^−1^ on sunny summer days (Bilger et al. 1995).

Chilling tolerance is commonly assumed to be the trait driving vigor under early planting, but this has not been formally tested, since isolating environmental variables under agronomically-relevant field conditions is challenging (Cooper et al. 2014). It is possible that some other stressor that we did not consider, such as mold, herbivores, or other unknown factors, might be influencing the field phenotype (Esele et al. 1993; Wu et al. 2019). Finally, reports of chilling-induced changes on root structure in maize (Richner et al. 1996) and relatively high levels of CBF expression in roots (Liu et al. 2013) suggest the possibility that *qCT04.62* regulates root-specific chilling tolerance, a tissue we did not directly investigate in this study.

### The potential for future studies of complex traits using Tan1 NILs

Controlled genetic studies using isogenic mutants or NILs are relatively rare in sorghum (Xin et al. 2021), making the NILs developed in this study a valuable genetic resource. There are several avenues of research on which these NILs could shed light. The NILs developed here can be useful to study pleiotropic functions of MBW ternary complex in cereals. Arabidopis *TTG1* has a broad regulatory function, which may be conserved in cereal orthologs of *TTG1* such as *Tan1*, maize *PAC1*, and rice *OsTTG1* (Tian and Wang 2020). Thus, if there are additional pleiotropic *tan1* loss-of-function phenotypes, studies of these *Tan1* NILs could elucidate unintended side effects that have been or should be, overcome with suppressor genes (Soyk et al. 2017).

Another potential use of the NIL is to study the adaptive role of proanthocyanidins. In plants, tannins are known to be important defense molecules to birds, fungi, and insect herbivores (Constabel et al. 2014). Proanthocyanidins are rare in cultivated grains as they are generally lost during domestication, and their adaptive role is not yet fully understood (Zhu 2019; Xie et al. 2019). Controlled experiments with these NILs could elucidate other agroecological effects of the multi-functional defense compounds and their role in adaptation.

## Material And methods

### Genetic analyses and plant materials

Data on published QTL was downloaded from the Sorghum QTL Atlas (Mace et al. 2019). QTL were filtered for biparental and NAM mapping studies and plotted by genomic location using custom R v4.1.2 scripts (R Core Team 2021). Three RILs from the chilling tolerant NAM BTx623 × Hong Ke Zi (PI 567946) family were used as starting material to reduce subsequent backcrossing effort (Marla et al. 2019). The RILs were then crossed to BTx623. F1 progeny were selected on two criteria: heterozygosity at the QTL of interest using a KASP marker system and visually for resemblance to BTx623, the recurrent parent. Selected progeny was then backcrossed to BTx623. Selection and backcrossing were repeated four times. Four suitable BC4F1 lines were then selected and selfed. From the segregating progeny, homozygotes for both alleles of the QTL of interest were selected, making eight total BC4F2 lines. Those eight lines were then advanced to the BC4F5 generation through single seed descent generating four pairs of NIL siblings (Marla et al. 2023).

### Genomic analyses

For whole-genome resequencing of NILs, leaf tissue was collected from two-week-old seedlings and frozen at -80 °C until DNA extractions. Following the manufacturer’s instructions, DNA extractions were performed using Quick-DNA Plant/Seed Miniprep Kit (ZYMO, D6020). DNA was quantified using a Thermo Scientific NanoDrop 2000/2000c Spectrophotometer. Library Preparation and DNA sequencing were performed by the Kansas State University Integrated Genomics Facility (https://www.k-state.edu/igenomics/index.html). DNA was sequenced to ~ 1 × depth on Illumina NextSeq 500 using 300 cycles and 151 paired-end chemistry.

Low-quality read sequences were trimmed using Trimmomatic v0.32 (Bolger et al. 2014), and the remaining reads were mapped to BTx623 v3.1.1 reference genome (McCormick et al. 2018) using BWA-MEM (Li 2013). Picard v2.26 MarkDuplicates was then used to merge bam files from common read groups and flag duplicate reads (Broad Institute 2019). SNPs were then called using GATK v4.2.5.0 suite of tools, including Haplotype Caller to create gVCF files, GenomicsDBImport to create gVCF database, and GenotypeGVCF to create final VCF (GA Van der Auwera and BD O’Connor 2020). BCFtools v1.15.1 was then used to sort variants and filter for high-quality biallelic SNPs (Danecek et al. 2021). A custom script was written using R v4.1.2 to analyze genome-wide sliding windows and plot alternate allele frequencies using 10,000 kb windows (R Core Team 2021). Two biological replicates were analyzed independently. Red is alternate^x^/alternate^HKZ^ >  = 0.2; blue is alternate^x^/alternate^HKZ^ < 0.2; yellow is when a color call differs between biological replicates.

### Grain tannin assays

The bleach test was performed as previously described (Waniska et al. 1992; Marla et al. 2019). Briefly, fifteen seeds from each genotype were placed in a 50-mL centrifuge tube. One mL of bleach/sodium hydroxide solution was added (3.75% NaOCl and 5% NaOH) to the seeds and left for 30 min. Seeds containing proanthocyanidins became dark, while non-proanthocyanidin seeds became white.

### Germination assays

Four temperature treatments were used to measure the genotypic effect on low-temperature germination, increasing from 10 °C to 25 °C in 5° increments, with three replicates per temperature. For each replicate, twelve seeds from each genotype were placed in a 90-mm Petri dish lined with filter paper and moistened with 2 mL distilled water. There were three Petri dishes per genotype, totaling 36 seeds per replicate. Dishes were sealed with parafilm and placed in a dark growth chamber at the treatment temperature. Each day for four days, Petri dishes were opened, visually inspected, and then documented with a photograph. Photographs were then scored for germination (Schneider et al. 2012) and analyzed using R v4.1.2 (R Core Team 2021). Graphs were created using ggplot2 v3.4.2 r package (Hadley Wickham 2016).

### Growth assays

The experiments were carried out in controlled environment chambers (Conviron Model CMP6050, Manitoba, Canada) at the Plant Growth Facilities at Colorado State University in Fort Collins, CO. Experiment designs were created and randomized using a custom R v4.1.2 script (R Core Team 2021). Each genotype/treatment combination had six replicates. Two temperature treatments were applied in parallel, chilling and control, in discrete growth chambers. For the long temperature treatment, control is defined as 30 °C/20 °C day/night temperature treatment and chilling 20 °C/10 °C. For the short temperature treatment control is defined as 28 °C/25 °C day/night temperature treatment and chilling 10 °C/4 °C. A consistent 12 h photoperiod and 700 μmol m^−2^ s^−1^ light intensity was used in both treatments.

Plants were potted in 1.5-inch Cone-tainers using Lambert LM-HP potting soil and given 3 g Osmocote controlled-release fertilizer. Water was provided in excess using a bottom watering system. For the long treatment, all pots were germinated under control temperature conditions for five days. Following germination, conditions for control plants remained unchanged, while chilling conditions were applied to chilling plants. After six weeks under treatment conditions, plant shoots were harvested, dried, and analyzed for dry weight. For the short treatment, all pots were germinated under control temperature conditions and grown for approximately seven days when chilling conditions were applied to chilling plants. After three days under treatment conditions, plants were again allowed to grow at control temperatures for seven more days. Plant shoots were then harvested, dried, and analyzed for dry weight.

### Photosynthetic assays

Experiment designs were created and randomized using a custom R v4.1.2 script (R Core Team 2021). Each genotype/treatment combination had six replicates. All plants were potted in 1.5-inch Cone-tainers using Lambert LM-HP potting soil and given 3 g Osmocote controlled-release fertilizer. Photoperiod was a 12 h day-night cycle with transits at 6:00 am and 6:00 pm. Light intensity was 700 μmol m^−2^ s^−1^, and water was provided in excess using a bottom watering system. Seedlings were allowed to grow at an optimal temperature until large enough for accurate leaf measurements to be taken for approximately ten days. Two temperature treatments were applied consecutively over a nine-day time course, optimal (28 °C/25 °C) and chilling (10 °C/4 °C) day/night. Throughout the time course, treatment changes occurred at 5:30 am on the scheduled day. The final day of the growth phase is day one for our time course analysis. Measurements were taken each day of the time course beginning at 10:00 am. On day two, seedlings were subjected to chilling treatment until day six. From day six through day nine, seedlings were again grown at optimal temperatures. Photosynthetic components were measured using MultiSpeQ (Kuhlgert et al. 2016) and analyzed using R v4.1.2 (R Core Team 2021). Graphs were constructed using ggplot2 v3.4.2 r package (Hadley Wickham 2016).

### Supplementary Information

Below is the link to the electronic supplementary material.

Supplementary file1 (DOCX 23 KB)

Supplementary file2 (DOCX 836 KB)

## Data Availability

Genetic stocks are available by request from the authors (Terry Felderhoff; tfelderhoff@ksu.edu) and will be submitted to the National Plant Germplasm System where they would be accessed via Germplasm Resources Information Network under SORGHUM-GENSTOCKS. Genomic and phenotype data are available at Dryad Data Repository (https://doi.org/10.5061/dryad.z34tmpgms).
