# Supplementary material for: Precise colocalization of sorghum’s major chilling tolerance locus with Tannin1 due to tight linkage drag rather than antagonistic pleiotropy
Source: Theor Appl Genet. 2024 Feb 3;137(2):42. doi: 10.1007/s00122-023-04534-4 (PMC10838249; doi:10.1007/s00122-023-04534-4)
Supplement: Supplementary file 1 — Supplementary file1 (DOCX 23 KB) [file 122_2023_4534_MOESM1_ESM.docx]

| **Table S1**. Summary of chilling tolerance QTL from joint linkage mapping in a nested association mapping population (Marla et al., 2019) visualized in Fig. 1. | | | |
| --- | --- | --- | --- |
| **Index** | **QTL** | **Chromosome** | **Position (v3.1)** |
| 1 | *qSbCT04.62* | Chr04 | 62,368,531 |
| 2 | *qSbEPEC_3-72* | Chr03 | 72,791,601 |
| 3 | *qSbCT02.08* | Chr02 | 8,672,301 |
| 4 | *qSbCT07.59* | Chr07 | 59,915,577 |
| 5 | *qSbCT09.57* | Chr09 | 58,070,153 |
| 6 | *qSbCT01.57* | Chr01 | 57,941,435 |
| 7 | *qSbCT01.06* | Chr01 | 5,730,743 |
| 8 | *qSbCT07.10* | Chr07 | 12,580,350 |
| 9 | *qSbEPSV1_3-01* | Chr03 | 1,447,612 |
| 10 | *qSbCT05.04* | Chr05 | 4,403,613 |
| 11 | *qSbCT01.13* | Chr01 | 13,526,795 |
| 12 | *qSbEPSV3_1-26* | Chr01 | 9,756,192 |
