# Supplementary material for: Precise colocalization of sorghum’s major chilling tolerance locus with Tannin1 due to tight linkage drag rather than antagonistic pleiotropy
Source: Theor Appl Genet. 2024 Feb 3;137(2):42. doi: 10.1007/s00122-023-04534-4 (PMC10838249; doi:10.1007/s00122-023-04534-4)

**Figure S1. SNP density across from genome resequencing for all sorghum lines**. Window size is 1 Mb. Dotted line is average genome wide SNP density.


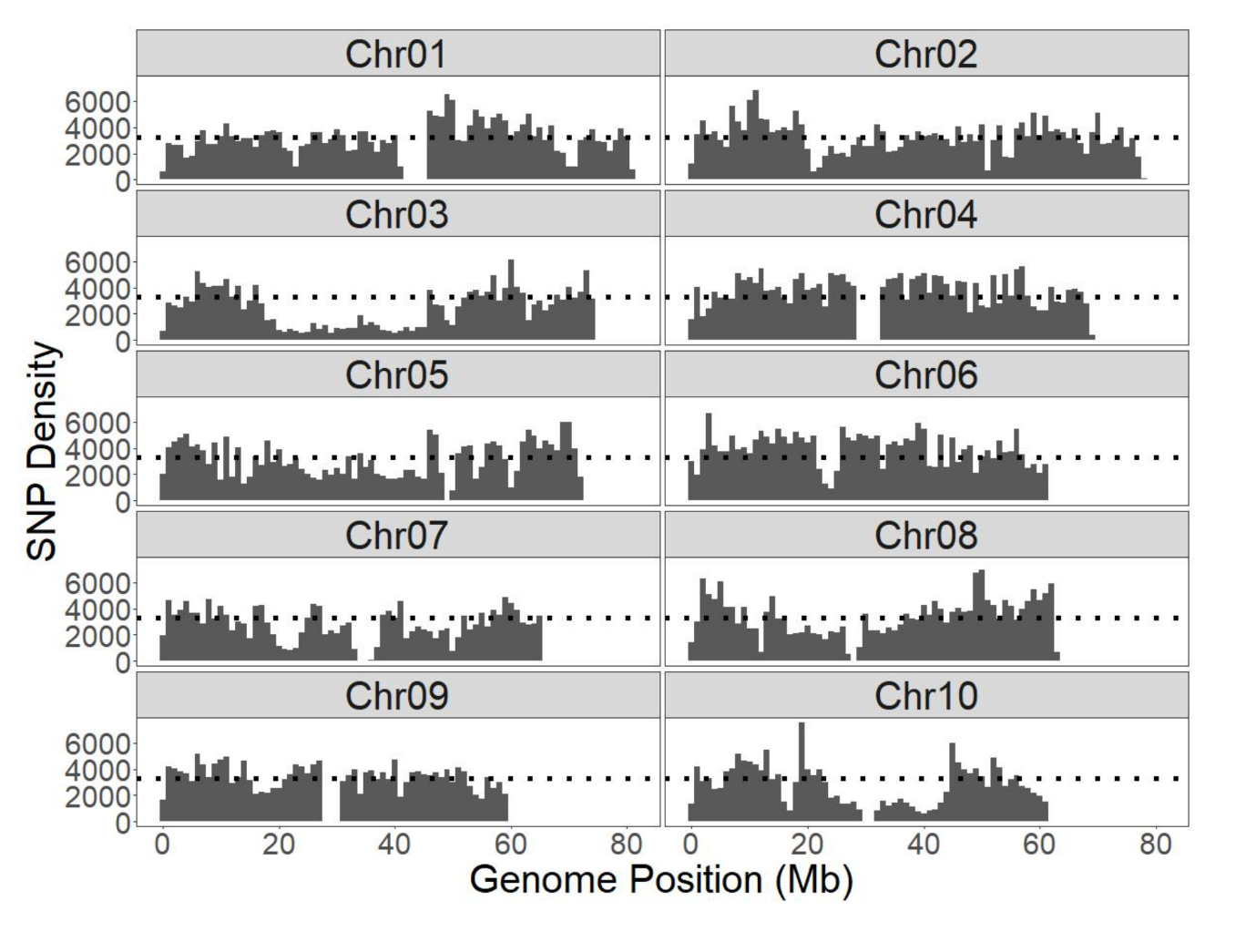

Supplement: Supplementary file 2 — Supplementary file2 (DOCX 836 KB) [file 122_2023_4534_MOESM2_ESM.docx]
